# Clinical Physicians’ Attitudes towards Evidence-Based Medicine (EBM) and Their Evidence-Based Practice (EBP) in Wuhan, China

**DOI:** 10.3390/ijerph16193758

**Published:** 2019-10-07

**Authors:** Jianan Hong, Jing Chen

**Affiliations:** School of Medicine and Health Management, Tongji Medical College, Huazhong University of Science & Technology, Wuhan 430000, China; m201875287@hust.edu.cn

**Keywords:** evidence-based medicine, evidence-based practice, clinical physicians, perception, attitude

## Abstract

*Objective:* Numerous studies have proved the importance of Evidence-Based Medicine (EBM) in daily clinical practice, however, clinicians’ attitudes play an important role in determining its implementation. The objective of this study was to investigate Chinese clinical physicians’ perception of and attitude towards EBM and their Evidence-Based Practice (EBP) as well as the barriers towards EBP. *Methods:* Using a cross-sectional design, self-response questionnaires were distributed to clinical physicians (internal medicine and surgery departments) across three tertiary hospitals in Wuhan, China. *Results:* In total, 131 out of 195 (67.2%) physicians completed and returned the questionnaire. A total of 64.9% of the physicians either knew moderately or a lot about EBM. The mean score of physicians’ attitude toward EBM was 2.35 ± 0.35, and that of their EBP skill/ competency was 1.51 ± 0.56 (on 0–3 Likert scale). In total, 76.0% of physicians often or sometimes applied EBM in routine daily practice. The largest barrier preventing implementation was the varying individual differences in diseases (61.0%), followed by a lack of investment from the hospital/department (39.8%), and a lack of patient cooperation (37.4%). Chinese physicians in tertiary hospitals possessed expressed positive attitudes towards EBM; however, they only retained a moderate level of clinical evidence competency. Both an individual factor (personal interest) and organizational factors (workload, hospital requirement) had an effect on physicians’ attitudes and their EBP skills. Management and organizational efforts, in addition to time dedicated for EBP projects could help reduce barriers that prevent EBP.

## 1. Introduction

Evidence-based medicine (EBM) is considered to be the careful and meticulous use of up-to-date evidence in the decision-making process regarding individual patient care, whereas evidence-based practice (EBP) integrates individual clinical expertise with the best available clinical evidence obtained from systematic research [1]. Since the term officially appeared for the first time in 1992 [1,2], EBM has been the focal point of medicine from both academic and professional perspectives, including EBP [3], evidence-based nursing [4], evidence-based health [5], evidence-based health policy [6], in research methods [7,8], in medical education and training [9,10], and during internship/residency [11]. The importance of EBM has been widely accepted by physicians such as physical therapists, pediatricians, dentists, and orthodontists. British and German surgeons rated EBM, on a scale from 1 to 10, as very important for daily clinical decision making (7.3), patients (7.8), and for the national health system (7.8) [12]. Moreover, Canadian family physicians embraced the facilitation of EBM in family practices and some physicians alluded to its significance in their daily implementation of EBM [13]. However, there is a great discrepancy between research evidence and the introduction of this evidence into routine clinical practice due to many factors, ranging from the quality of the evidence, health professionals, patients, and health institutions to healthcare systems [14,15]. Moreover, developing countries encounter more difficulties and challenges related to EBM implementation and practice [16].

The official introduction of EBM into China can be traced back to the year 1996, when The Chinese Cochrane Center (The Center) was established. The Center was approved by China’s former Health Ministry in 1997 and became the 14^th^ center of the International Cochrane Collaboration in 1999. EBM then spread rapidly across China through the introduction of Clinical Epidemiology and EBM as compulsory curricula in medical universities, as well as through the launch of academic journals such as the *Chinese Journal of Evidence-Based Medicine* (2001),the *Journal of Evidence-Based Medicine* (2001), the *Chinese Journal of Evidence-Based Pediatrics* (2006), the *Chinese Journal of Evidence-Based Cardiovascular Medicine* (2008), and through the establishment of professional institutions and academic organizations, such as the *Chinese Medical Doctors Association’s Evidence-Based Medicine Committee* (2003) and the *Ministry of Education’s Virtual Research Center of Evidence-based Medicine* (2004) [17,18].

Although an increasing number of empirical studies on physicians’ knowledge, perception, attitude, competency, and practice of EBM in clinical practice settings have been witnessed in recent years, the relationship between knowledge/attitude and behavior has not been fully explored. Most empirical studies have been conducted in developed countries and few studies have been carried out in developing countries; additionally, very little English literature focuses on Chinese physicians [19]. In this study, we investigated physicians’ knowledge of, and attitudes towards EBM and their EBP skills and competency, as well as the influencing factors and barriers towards EBP among Chinese clinical physicians. 

## 2. Materials and Methods

This study employed a cross-sectional survey design. The 2013 China Health Statistical Yearbook reported that there were 2,138,836 medical practitioners in China, 1,297,078 of whom were working in hospitals, with 74.1% (961,018) of them in general hospitals and 1.4% (18,379) in TCM-WM (Traditional Chinese Medicine-Western Medicine) hospitals. Based on the above data and a convenience sampling method, three tertiary public hospitals in the city of Wuhan located in Central China were chosen. Hospital A is a general and teaching hospital, Hospital B is a TCM-WM and teaching hospital, and Hospital C is a general and non-teaching hospital. Tertiary public hospitals were chosen because they are the highest-level hospital in China’s medical delivery system. Physicians in these hospitals are more likely to be highly trained and more educated compared to those in secondary and primary hospitals. 

The target population was hospital physicians and resident physicians, however, refresher physicians were excluded. Subjects from each hospital were recruited mainly from two departments (Internal Medicine and Surgery). 

Hospital administrators and/or the directors of the departments distributed the questionnaires to physicians. Completed questionnaires were recovered by the distributor and checked by the researchers for quality of data collection. The contact telephone number of one of the researchers was included in the cover letter with the questionnaires in case the physicians needed to ask questions about the study. In total, 195 questionnaires were sent out to physicians and 131 completed questionnaires were returned. Demographic data are presented in Table 1.

### 2.1. Questionnaire

Based on the definitions of EBM and EBP and relevant references such as McColl (1998) [20], Heiwe et al. (2011) [21], and Lammers et al. (2011) [22], the instrument used in this study was developed. The first section of the questionnaire focused on the participant’s knowledge of, and attitudes towards EBM (6 items). Each item was rated on a four-point scale: ‘0 = none/unimportant’, ‘1 = a little’, ‘2 = moderately’ and ‘3 = very’. The second section was designed to determine the physician’s skills (8 items) and EBP competency (4 items). Using a scale of 0 to 3 (‘0 = none’, ‘1 = some’, ‘2 = moderate’ and ‘3 = a lot’), physicians’ skills were measured by assessing the extent to which respondents knew about professional terminology. EBP competency was comprised of how often the physician applied EBP and the extent to which they considered factors of the decision-making process. This was measured on a four-point scale (‘0 = none/never’, ‘1 = some/rarely’, ‘2 = moderate/sometimes’ and ‘3 = much/often’). The third part of the questionnaire was developed to investigate the physician’s perceived barriers towards EBP implementation. 

### 2.2. Data Analysis

Statistical analyses were performed using IBM, SPSS version 13.0 (SPSS Inc., Chicago, IL, USA). Descriptive statistics were used to examine the frequencies of the respondents’ demographics, as well as the physicians’ opinions about EBM and EBP. Univariate and multivariate analyses analyzed the factors influencing physicians’ knowledge of, and attitudes towards EBM, as well as their skills/competency. Linear regression models were used to identify the factors that affected physicians’ attitudes towards EBM and their EBP skills/competence. Logistic regression models were used to identify the factors with a significant effect on physicians’ knowledge of EBM and the implementation of EBP.

## 3. Results

### 3.1. Demographic Characteristics

In total, 131 out of 195 (67.2%) physicians completed and returned the questionnaire. However, the questionnaire was incompletely filled. Table 1 displays the demographic details. 

### 3.2. Physicians’ Knowledge and Perception of EBM

Most (58.0%) physicians knew moderately about EBM, while only 2.3% of them knew nothing about EBM. Physicians learned about EBM through a variety of channels. The main channel through which physicians learned about EBM was continuing education (61.8%), followed by school education (54.2%) and hardcopy journals (51.9%), see Table 2. 

Table 3 illustrates physicians’ perceptions on the importance of clinical evidence (96.1%), patients’ will (90.6%), and personal skill/experience (96.9%) in the decision-making process. Among all demographic variables, only specialties served as a statistically significant predictor for physicians’ knowledge of EBM in univariate logistic regression models. Internists were more likely to report that they knew a lot or moderately about EBM than surgeons. The odds ratio for this association was 4.327. 

### 3.3. Physicians’ Attitudes towards EBM

The mean score of physicians’ attitudes toward EBM was 2.35 ± 0.35 (on a 0–3 Likert scale). The univariate linear regression investigating attitudes towards EBM found gender, specialty, number of hours worked per week, hospital’s scientific research requirement, and a physician’s personal interest in scientific research to be statistically significant predictors. However, when those variables were entered into a multivariate linear regression model, only physicians’ personal interest in scientific research (Beta = 0.249, *p* = 0.006), specialty (Beta = 0.221, *p* = 0.014), and the number of working hours per week (Beta = 0.206, *p* = 0.022) still had a significant association with physicians’ attitudes, which explained 13.2% of physicians’ attitudes towards EBM (Table 4).

The most important reasons why physicians had a willingness to learn EBM was their desire to facilitate clinical practice (79.2%) and academic research (78.5%), followed by a teaching demand (22.3%), and hospital arrangement (8.5%).

### 3.4. Physicians’ Skills and EBP Competency

Table 5 illustrates how much physicians knew about professional terms. The mean score of 12 items was 1.51 ± 0.56 (on 0–3 point Likert scale), which indicates that physicians did not have enough skill/competency. 

Among gender, educational background, specialty, attitude, the hospital’s requirement and personal interest, attitude, educational background and a hospital’s research requirement accounted for 22.9% of the physicians’ skills/competence (Table 4). 

### 3.5. Physicians’ EBP Behavior and the Barriers towards EBP

This study indicates that 17.7% and 58.5% of physicians, respectively, often and sometimes applied EBM in routine daily practice, and that 22.3% and 1.5% of physicians, respectively, rarely and never applied. Specialty and EBP skills/competency explained the 26.3% of physicians who implemented EBP (Table 4). 

As for the barriers towards the implementation of EBP (Table 6), 61.0% of physicians cited that the largest barrier was the varying individual differences in diseases, followed by a lack of investment from the hospital or department (39.8%), and uncooperative patients (37.4%).

## 4. Discussion

We believe that our investigation is an important effort to assess Chinese clinical physicians’ perception of, and attitudes towards EBM and their behaviors related to EBP, as well as the barriers that impede EBP. Firstly, Chinese physicians in this study possessed moderate levels of EBM knowledge and positive attitudes towards EBM, which is at a similar level to that of their counterparts in Britain, Germany [12], France and Switzerland [23].

The establishment of the Chinese Cochrane Center in 1996 was a symbol of the official introduction of EBM into China. After that, a new development model integrating discipline, platform, echelon and popularity [24] was adopted in view of the fact that Chinese clinical professionals and medical students did not have sufficient training for health technology assessment, epidemiology, and evidence-based medicine. Many Western countries may simply rely on the National Cochrane Center for research, training, and academic activities, however, in China, EBM training and research basically relied on universities [25], with approval of the National Ministry of Education, which firmly developed EBM discipline, established an EBM platform, and created an EBM echelon. With the initial target of medical students [13], EBM developed rapidly in universities, and EBM academic organizations and seminars on national, provincial, and municipal levels and EBM academic journals promoted EBM much further.

As expected, here was no strong collision between evidence-based medicine and traditional Chinese medicine or perhaps EBM was only blocked at the individual level of traditional Chinese medicine, owing to the top-down policy path in China. On the contrary, EBM has also burgeoned in the field of traditional Chinese medicine, with the evidence-based Medicine Center of Chinese Traditional Medicine being established [26] in 2019.

In fact, EBM has been disseminated throughout China during the last two decades. Support from the Chinese government and its relevant departments, such as China’s former Health Ministry, the National Ministry of Education, and the National Natural Science Foundation [27], may have played a significant role in promoting Chinese physicians’ perception of, and attitudes towards EBM.

However, although physicians had positive attitudes towards EBM, their clinical evidence competence was just at a moderate level, and the actual execution of EBP was not as frequent as expected. This continues to be an abstract field, which, although recognized as important, is not always used in daily clinical practice [28]. There exists a complex process that can be affected by a mix of factors from physicians’ attitudes to EBP skills to the implementation of EBP.

Our results reveal that physicians’ attitudes towards EBM could be influenced by both an individual factor (interest in scientific research) and an organizational factor (workload). Interest, as an intrinsic motivation and its creation and maintenance is an important tool during learning and instruction [29]. It can be said that the emergence of physicians’ interest is the cornerstone of their positive attitudes and the first step to conduct EBP. In terms of workload, time is a vital guarantee for EBP and naturally, physicians who own less personal time spend less time training and interpreting evidence [20].

In addition, we found that physicians’ EBP skills/competence could be influenced by both an individual factor (attitudes) and an organizational factor (hospital’s scientific research requirement). The relationship between attitudes and skills has also been found among registered nurses in China [30]. At the same time, external research requirements, as a mandatory role, can impel physicians to improve their skills to achieve EBP as well. A previous study about nurses [31] also demonstrated that a significant factor in promoting EBP was organizational management, for example, support from the hospital to use and conduct research.

This study shows that physicians’ EBP competence affected their implementation of EBP, which is consistent with the findings of a study in the USA [32] that the development of evidence-based competence provided a key mechanism for the implementation of EBP.

Lastly, time is usually the foremost-cited barrier to the implementation of EBP in many studies [14,16,33,34], yet, its importance ranked fifth in Chinese samples. The largest barrier in this study was the varying individual differences in diseases. This may well be due to the phenomenon that the overall quality of clinical guidelines in China was generally low, nevertheless, the quantity was large [35], which casts Chinese physicians’ uncertainty and doubt on the rationality and science of existing evidence. As a result, it is urgent to develop national clinical guidelines of high quality and routinely incorporate them into EBP. Additionally, the results indicate that the second and third barriers were lack of investment from the hospital/department, followed by a lack of patient cooperation. A Swedish study has found that patient participation and patient preferences affected the actual use of evidence in clinical practice [21]. The premise of EBP is the choice of patients to get treatments that are evidence-based. Our results also imply that it is significant to make a sufficient investment. This may be owing to the extensive impact of EBP on other factors relevant to finding and reviewing evidence, such as EBP resources and training. Previous studies concerning nurses [36,37,38] also found that comparing with barriers to changing practice, those relating to finding and reviewing evidence were more problematic. It is important for healthcare staff to be supported when conducting EBP [35].

This study has several limitations. First, we used a small convenience sample that focused on one city in China. A total of 32% of physicians did not complete the questionnaire; we could not identify how many of them were a missing sample and how many of them were EBM-minded. Secondly, since Chinese physicians in different types of hospitals (such as tertiary hospital or community health-service centers) in various cities may respond differently, the results were representative of physicians in tertiary hospitals, not representative of physicians in secondary and primary hospitals. Thirdly, we concentrated on individuals and the hospital level. Further studies are needed to explore patients’ perception of, and attitudes towards EBM, which can influence the design of EBM education and the implementation of EBP.

## 5. Conclusions

Overall, this study shows that Chinese physicians possessed expressed positive attitudes towards EBM; however, they only retained a moderate level of clinical evidence competency. Both an individual factor (personal interest) and organizational factors (workload, hospital requirement) had an effect on physicians’ attitudes and EBP skills. The first three barriers preventing EBP were the varying individual differences in diseases, a lack of investment from the hospital/department, and a lack of patient cooperation. It is important to develop national clinical guidelines of high quality and routinely incorporate them into EBP. Management and organizational efforts in addition to time dedicated for EBP projects could help reduce barriers that prevent EBP.

## Figures and Tables

**Table 1:** Characteristics of the 131 Survey Respondents.

Characteristics	No./Total No.	Physicians (%)
**1. Gender**		
Male	67/128	52.3
Female	61/128	47.7
**2. Age**		
24 year	49/125	39.2
31 year~	53/125	42.4
36 year~50 year	23/125	18.4
**3. Educational background**		
Junior college	1/129	0.8
Bachelor’s degree	56/129	43.4
Master’s or Doctoral degree	72/129	55.8
**4. Technical title**		
To be assessed	8/120	6.7
Junior	54/120	45.0
Middle	45/120	37.5
Senior	13/120	10.8
**5. Professional specialty**		
Internal medicine	42/127	33.1
Surgery	75/127	59.1
Others	10/127	7.9
**6. Number of years working**		
1 year~	52/131	41.6
6 year~	41/131	32.8
11 year~30 year	32/131	25.6
**7. Working hours per week**		
Below 60 h	49/128	38.28
61 h~70 h	43/128	33.59
Above 70 h	36/128	28.13
**8. Hospital**		
A	50/131	38.2
B	46/131	35.1
C	35/131	26.7
**9. Hospital’s scientific research requirement**		
Low	19/127	15.0
Moderate	72/127	56.7
High	36/127	28.3
**10. Personal interest in scientific research**		
Low	23/129	18.3
Moderate	84/129	66.7
High	19/129	15.1

**Table 2:** Physicians’ knowledge and learning channels of Evidence-Based Medicine (EBM).

Item		No./Total No.	Physicians (%)
Knowledge of EBM	Nothing	3/131	2.3
	A little	43/131	32.8
	Moderate	76/131	58.0
	A lot	9/131	6.9
EBM learning channels	Continuing education	81/391	61.8
	School education	71/391	54.2
	Hardcopy journals	68/391	51.9
	Internet	57/391	43.5
	Colleagues	45/391	34.4
	Hospital arrangement/propagation	36/391	27.5
	Advanced training	31/391	23.7

**Table 3:** Physicians’ perceptions of and attitudes towards EBM (No./Total No.).

	Item	Somewhat	Quite	Very
Importance of the three factors of EBM	Scientific evidence	5/129	58/129	66/129
	Patient’s will	10/128	79/128	37/128
	Personal skill/experience	4/128	68/128	56/128
Attitudes	Importance of applying EBM	1/130	80/130	49/130
	Importance of evaluating literature	4/130	77/130	47/130
	Usefulness of medical literature in routine practice	5/131	80/131	46/131
	Willingness to learn EBM	1/131	75/131	55/131

**Table 4:** Multivariate predictors of physicians’ attitudes towards EBM, competence of EBP, and the implementation of EBP.

Dependent Variable	Predictors	Beta	T	*p* Value	B (95% CI)	OR (95% CI)	**R Square**
Attitudes towards EBM	(constant)		18.038	0.000	(1.790–2.232)		0.132
	Interest (high) *	0.249	2.810	0.006	(0.067–0.390)		
	Specialties (internist) *	0.221	2.498	0.014	(0.033–0.285)		
	Working hour per week (above 70 h) *	0.206	2.329	0.022	(0.024–0.297)		
EBP skill/competence	(constant)		−0.006	0.995	(−0.741–0.736)		0.229
	Hospital’s scientific research requirement*	0.266	2.946	0.004	(0.091–0.467)		
	Attitude towards EBM	0.266	3.026	0.003	(0.131–0.631)		
	Educational background (Master’s or Doctoral degree) *	0.217	2.470	0.015	(0.043–0.389)		
EBP #	Specialties (internist) *	7.397	1	0.007		(1.528–13.604)	0.263
	EBP skill/competence	5.134	1	0.023		(1.215–14.766)	
	(constant)	7.542	1	0.006			

* Contrast group: Interest (moderate or low), specialties (surgeon), working hour per week (below 70 h), hospital’s scientific research requirement (moderate or low), and educational background (Bachelor’s degree below). #: logistic regression model was used. 0 = never/rarely applying EBM, 1 = sometimes/often applying EBM.

**Table 5:** Physicians’ EBP skills and competence (No./Total No.).

	Item	None	A Little	Some	A Lot
EBP skills	Clinic Epidemiology	9/129	94/129	21/129	5/129
	Medical Statistics	21/129	77/129	26/129	5/129
	Medical English	15/129	80/129	27/129	7/129
	Medical Information Retrieval	10/129	75/129	35/129	9/129
Terms	Randomized controlled trial (RCT)	4/128	29/128	57/128	38/128
	META analysis	19/127	54/127	40/127	14/127
	Bias	12/127	46/127	45/127	24/127
	Random sampling	4/128	20/128	65/128	39/128
	Homogeneity	22/125	52/125	44/125	7/125
	P value	5/127	33/127	65/127	24/127
	Confidence interval	10/126	49/126	55/126	12/126
	Relative risk (RR)	7/126	57/126	53/126	9/126

**Table 6:** Physicians’ barriers to the implementation of EBP (%).

Rank	Perceived Barriers of EBP	Frequency	Percentage
1	Varying individual differences in diseases	75	61.0
2	Lack of investment from hospital or department	49	39.8
3	Lack of patient cooperation	46	37.4
4	The simplicity of decision-making	42	34.1
5	Limited time	40	32.5
6	Lack of supportive culture/climate of hospital or department	29	23.6
7	Rapidly updated medical technology	28	22.8
8	Lack of relative proficiency	26	21.1
9	Lack of supportive management institution in hospital or department	21	17.1
10	Lack of interest	5	4.1
